# The Brazilian National Oral Health Policy and oral cancer mortality trends: An autoregressive integrated moving average (ARIMA) model

**DOI:** 10.1371/journal.pone.0291609

**Published:** 2023-09-21

**Authors:** Elisa Miranda Costa, Elisa Santos Magalhães Rodrigues, Francenilde Silva de Sousa, Felipe Bezerra Pimentel, Mariana Borges Sodré Lopes, João Ricardo Nickenig Vissoci, Erika Barbara Abreu Fonseca Thomaz

**Affiliations:** 1 Public Health Department, Federal University of Maranhão, São Luís, Maranhão, Brazil; 2 Department of Electrical Engineering, Federal University of Maranhão, São Luís, Maranhão, Brazil; 3 Duke Global Health Institute, Duke University, Durham, North Carolina, United States of America; Lithuanian University of Health Sciences, LITHUANIA

## Abstract

**Objective:**

This study analyzes the effect of the Brazilian National Oral Health Policy (NOHP) on oral cancer mortality rates (OCMR).

**Method:**

This is an ecological study with secondary oral cancer death data, using interrupted time series analysis (ARIMA, Autoregressive Integrated Moving Average). Annual death data were collected from the Mortality Information System (1996–2019). The outcome was the OCMR, standardized by gender and age We considered the NOHP, categorized as “0” (before its implementation), from 1996 to 2004, and “1 to 15”, from 2005 to 2019. ARIMA modeling was carried out for temporal analysis, and regression coefficient estimation (RC).

**Results:**

The Brazilian NOHP implementation was associated with an increase in OCMR in the North region (CR = 0.16; p = 0.022) and with a decrease in the Southeast region (CR = -0.04; p<0.001), but did not affect the other macro-regions nor Brazil. The forecast models estimated an increase in OCMR for the North, and Northeast, a decrease for the Southeast, and stability for the South and Brazil.

**Conclusion:**

The Brazilian NOHP is not being effective in reducing the OCMR. The trends behaved differently in the Brazilian territory, highlighting health inequities. We recommend that the NOHP strengthen the oral health care network, incorporating oral cancer as a notifiable disease, adopting strategies for prevention, screening, and providing opportunities for early treatment of the disease.

## Introduction

According to the 2019 Global Burden of Disease Study, oral and oropharyngeal cancers accounted for approximately 3.1% of deaths from all cancers in the world in 2019, which represents about 313,000 deaths [1]. In 2019, Brazil had a standardized oral cancer mortality rate (OCMR) of 3.01 per 100,000 inhabitants, 5.1 for men, and 1.22 for women [2]. When evaluating the historical series between 2002 and 2013, the rate increased in the Northeast (6.9%) and decreased in the Southeast (-2.9%), with a stationary behavior for the other regions [3]. From 2000 to 2013, the annual rate trend was stationary for men and increasing for women (1.31%), with higher growth patterns for brown men (20.36%) and women (8.24%) [4].

The high OCMR may reflect a lower Human Development Index, lower investment in health services [5, 6], and delayed diagnosis and onset of treatment in more advanced clinical stages [7, 8]. Besides, the greater coverage of oral health services in primary health care (PHC) [5, 6, 8–11] and a higher number of Dental Specialty Centers (acronym in Portuguese, CEO) [10] are associated with lower OCMR. These results point to the importance of expanding the coverage of services at each point of the Health Care Network (HCN).

In 2004, the NOHP promoted the expansion of oral health teams in PHC, with the potential to carry out an active search for cases in the territory, and stimulated the implementation of CEO, with a mandatory provision of services for oral cancer diagnosis [12]. Also, NOHP has a national scope, interfacing with other policies, and including different health services systems components, such as infrastructure (facilities, equipment, medicines, and knowledge) and human resources (health workers), financing (financial contribution to the program in the federal budget, Social Security, social insurance, and private insurance), management and organization (organization of services and networks and implementation of actions and programs), and care model (how the provision of services and the work processes of health professionals are carried out) [13]. Therefore, we hypothesize that NOHP can affect the OCMR. However, there is a lack of evidence on whether the implementation of the NOHP in 2004 impacted the Brazilian OCMR trends in the last 15 years.

There are some studies on the trend of OCMR by regions [3, 4], but more current analyses, considering the pre and post-NOHP period, are scarce. We identified three studies analyzing the effect of some components of NOHP on OCMR. However, they did not include the pre-NOHP period [10] or did so for a very short period [9, 11]; they did not consider the stationarity and interruption components of the series; nor did they make effect assessments by macro-region [10, 11].

Therefore, to our knowledge, this is the first interrupted time series analysis with national OCMR data in Brazil using ARIMA (autoregressive integrated moving average), a popular model in the econometric field that allows analyzing the behavior of stationary and non-stationary series, and the effect of programs and policies on specific outcomes over time [14, 15]. The ARIMA model with input series is called an intervention model or interrupted time series model [14, 15]. In an intervention model, the input series is an indicator variable that contains discrete values that flag the occurrence of an event affecting the response series [14, 15]. This event is an intervention in or an interruption of the normal evolution of the response time series [14, 15]. This method has already been applied to investigate the impact of COVID-19 on dental service production indicators in a Brazilian state, for example [16].

Moreover, analyzing the effects of this policy over space-time in low- and middle-income countries context can assist us in planning and formulating strategies for more equitable health services regarding prevention, early diagnosis, treatment, and rehabilitation. This paper aims to analyze the temporal trends of OCMR for Brazil and macro-regions, and estimate the effect of NOHP on the OCMR.

## Materials and methods

### Study design and study area

This ecological, interrupted time series study [14] followed the STROBE statement. The units of analysis were the Brazilian macro-regions and Brazil. Brazil is the largest country of Latin America, with a Gross Domestic Product (GDP) per capita equal to 5,521 billion reais, and a Gini Index of household income per capita equal to 0.6086. Brazil is composed of 26 states and the Federal District, grouped into five regions with great socio-economic inequalities. The North and Northeast regions have a greater territorial extension, but are less populous and have a lower GDP. The Midwest, except the Federal District, also presents indicators of lower development. On the other hand, the Southeast and South regions are the most populous, the richest, with the most organized HCN and the best development indicators.

### Ethics statement

This study was based on secondary data from Brazilian public domain databases, with no need to be approved by an Ethics Committee, according to Resolution No. 510/2016 of the National Board of Health.

### Outcome and data availability

The time-dependent variable for this study was the OCMR, standardized by gender and age using the direct standardization technique. Oral cancer deaths were collected and classified according to the International Classification of Diseases (ICD-10), considering codes C-00 to C-10, from 1996 to 2019. The number of oral cancer deaths was collected from the Mortality Information System (acronym in Portuguese, SIM), openly available at https://datasus.saude.gov.br/transferencia-de-arquivos/. The denominator was the total population obtained from the Brazilian Institute of Geography and Statistics (acronym in Portuguese, IBGE), considering the 1991, 2000, and 2010 censuses, and inter-census projections. The data referring to the Brazilian population were gathered from the DATASUS, available at https://datasus.saude.gov.br/populacao-residente. For this study, we calculated the OCMR for every 100 thousand inhabitants.

### Intervention or exogenous variable

The NOHP was the exogenous variable of this study. An intervention should meet the following criteria to be chosen in ARIMA models: it should be an action that can affect the outcome and a nationwide action if this is the unit of analysis [15]. In this sense, the document with the NOHP guidelines, which should be implemented throughout the national territory to expand coverage and access to oral health services, was published in 2004. This policy has guiding principles for the reorientation of oral health care models centered on PHC, expanded specialized services in the CEO and hospitals, primarily focused on diagnosis, treatment, and rehabilitation of oral cancer patients. Some evidence shows that the PNSB (mainly due to the expanded coverage of the Family Health Strategy–ESF) affects the reduction of dental caries [17] and the reduction in the percentage of individuals who never had a dentist [18]. PNSB’s effects on oral cancer indicators are still hardly studied [11, 19].

### Exploratory analysis

The decomposition of the series was conducted to study the trend and residual characteristics [14]. Seasonality analysis, however, was not considered as the time cycle was annual. The Prais-Winsten regression, which predicts first-order autocorrelation correction [20], was used to calculate the annual percentage change (APC) of the OCMR. The increase, decline, and stagnation trends were expressed as APC and considered stationary when the regression coefficient did not differ from zero (p>0.05).

### ARIMA for the intervention effect analysis

This model is based on adjusting the observed values to reduce the difference between the values produced in the predicted model and the observed values to close to zero. This model can describe the behavior of stationary and non-stationary series. The most general form of the non-seasonal model is the ARIMA (p,d,q), where AR: (p = degree of the autoregressive part); d = degree of the first difference involved) and MA: (q = degree of the moving average part). The Box-Jenkins method for time series includes three steps: 1) Identification (Data Preparation and Model Identification); 2) Estimation and Testing; (Estimation and Diagnosis), and 3) Application (Prediction and Health Intervention) [15].

Data preparation included applying the stationarity test (*ur*.*kpss*) and estimating the number of differences (*ndiffs*). Identifying the ARIMA model started manually by estimating the p and q operators, which occurred from the assessment of data autocorrelation (ACF) and partial autocorrelation (PACF). The ACF identifies how much the information from the previous period is related to the following observation. The PACF evaluates the degree of correction of the current variable with its previous values while keeping the other values constant. The identification process was also automated by the *auto*.*arima* function, which indicates the best-fit model, considering a variation of the algorithm that combines unit root tests, Akaike’s Information Corrected Criterion (AICc) minimization, and Maximum Likelihood Function (MLF) to obtain an ARIMA model. The smaller the value of the criterion, the better adjusted the model was; in other words, the model achieved the best adherence to the observed data [14, 15].

The following potential parameters were considered to diagnose the appropriate model to perform the forecast: i) RMSE (root mean squared error); ii) MAE (mean absolute error); and iii) MAPE (mean absolute percentage error). After estimating these parameters, the parsimony criterion was adopted to select the model with the fewest possible parameters and suitable for an adequate representation of the observed series. We also observed normality and autocorrelation between residuals.

The model application phase included the analysis of the effect of the NOHP on the OCMR. The intervention was addressed as a numerical variable, in which the value “0” corresponds to the pre-NOHP implementation years (1996–2004), and increasing values from 1 to 15 correspond to post-NOHP intervention years (2005–2019). The ARIMA model selected during the identification stage was adopted and applied, including the NOHP intervention. The regression coefficient (RC) values were evaluated to assess the intervention’s effect. The model adequacy was adopted in the absence of spikes, autocorrelation, and partial autocorrelation, and application of the Box-Pierce test (alpha = 5%). We conducted analyses using RStudio version 4.2.0 software.

## Results

**Fig 1:**
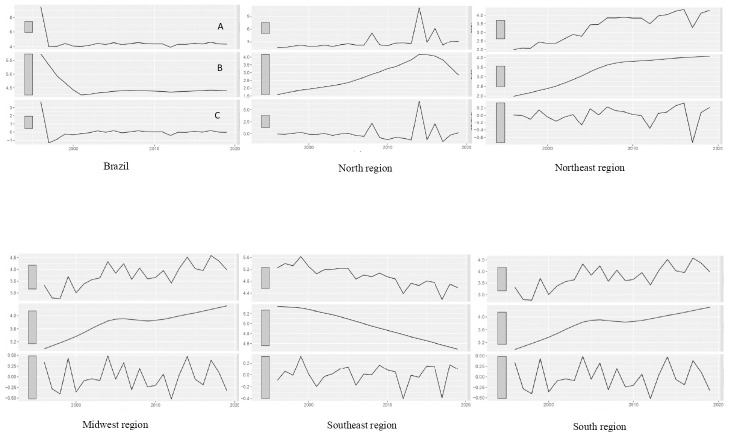
Decomposition of the time series by macro-regions and Brazil, 1996–2019. A–Observed Series B–Trend extracted from the series C—Series formed by the residuals.

OCMR had an increasing trend in the North, Northeast, and Midwest, a decreasing trend in the Southeast, and a stable trend in the South and Brazil (Table 1, Fig 1). We carried out one differentiation for the North, Northeast, Southeast, and Midwest non-stationary series. The South and Brazil did not require differentiation due to their stationary behavior.

**Table 1:** The trend in oral and oropharyngeal cancer standardized mortality rates, and annual percentage rate change (APC) by macro-region, Brazil, 1996–2019.

Unit of Analysis	Coefficient	95%CI^a^	P-Value	R^2^^b^	APC^c^	Trend
North	0.128	0.036 to 0.220	0.009	25.92	34.43	Growing
Northeast	0.100	0.069 to 0.130	0.000	55.66	25.90	Growing
Southeast	-0.041	-0.050 to -0.032	0.000	79.63	-9.13	Decreasing
South	0.012	-0.031 to 0.057	0.563	35.3	2.945	Stable
Midwest	0.049	0.028 to 0.071	0.000	50.73	12.18	Growing
Brazil	-0.002	-.0062 to 0.001	0.296	71.03	-0.48	Stable

^A^ 95%CI: 95% Confidence Interval.

^B^ R^2^: Adjusted R squared.

^c^APC: Annual Percentage Change

**Fig 2:**
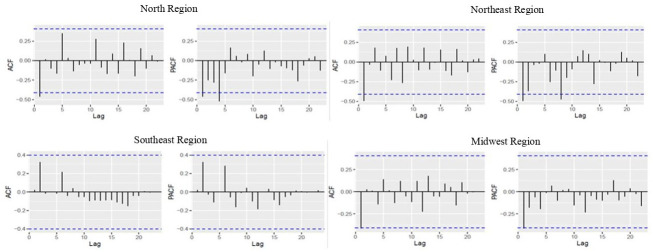
Graphs of the autocorrelation and partial autocorrelation functions after differentiation for selecting the ARIMA model, time series by macro-regions and Brazil, 1996–2019.

The ARIMA models with the best fit were: (0,1,1) for North and Northeast; (4,1,0) for Southeast; (0,1,2) for Midwest; (0,0,0) for South and Brazil as they had stationary series, and there was no correlation between the lags (Table 2). After the series differentiation, we observed a decrease in spikes when estimating the autocorrelation and partial autocorrelation of the North, Northeast, Southeast, and Midwest. The residuals did not show autocorrelation and there was no significant peak in any lag, so the residuals were uncorrelated for the macro-regions and Brazil. Additionally, when applying the Ljung-Box Test, p-values were higher than 0.05 for all the time series: North (p = 0.081); Northeast (p = 0.880); Southeast (p = 0.066); Midwest (p = 0.455); South (p = 0.949); Brazil (p = 0.980) (Fig 2). The parameters of the models and accuracy measures were adequate. Furthermore, the residuals did not correlate with each other since the p-values from the Box-Pierce Test were higher than 0.05 for all Brazilian macro-regions and Brazil.

**Table 2:** Parameter estimates for selecting the ARIMA model, time series by macro-regions and Brazil, 1996–2019.

Unit of Analysis	Parameters	Log-L^a^	AIC^b^	AICc^c^	BIC^d^	RMSE^e^	MAE^f^	MAPE^g^
North	ARIMA (0,1,1)	- 47.85	101.71	102.97	105.11	1.77	0.99	29.23
Northeast	ARIMA (0,1,1)	- 6.02	18.05	19.31	21.46	0.30	0.19	5.71
Southeast	ARIMA (4,1,0)	10.89	- 9.79	- 4.54	- 2.97	0.13	0.09	1.99
Midwest	ARIMA (0,1,2)	- 10.47	26.93	28.2	30.34	0.36	0.27	7.46
South	ARIMA (0,0,0)	- 29.34	62.69	63.26	65.04	0.82	0.39	8.11
Brazil	ARIMA (0,0,0)	- 34.97	73.94	74.51	76.3	1.03	0.41	9.12

^a^AIC: Akaike Information Criterion

^b^AICc: Akaike Information Corrected Criterion

^c^BIC: Bayesian Information Criterion

^d^RMSE: Root Mean Square Error.

^e^MAE: Mean Absolute Error

^f^MAPE: Mean Absolute Percentage Error

The implementation of the NOHP was associated with an increased OCMR in the North (RC = 0.16; p = 0.022) and a decrease in the Southeast (RC = -0.04; p<0.001), but did not have any effect on OCMR over time in the other macro-regions and Brazil (Table 3).

**Table 3:** Relationship between the implementation of the Brazil National Oral Health Policy on the oral cancer mortality rate trends in the Brazilian macro-regions and Brazil, 1996–2019.

Unit of Analysis	Parameters	Log-L^a^	AIC^b^	AICc^c^	BIC^d^	RMSE^e^	MAE^f^	MAPE^g^	Coef.^h^	P-Value
North	ARIMA (0,1,1) with drift	-47.98	101.95	103.22	105.36	1.78	0.99	26.60	**0.16**	**0.022**
Northeast	ARIMA (0,1,1) with drift	-7.48	20.96	22.22	24.37	0.33	0.22	6.54	0.09	0.143
Southeast	ARIMA (4,1,0)	7.99	-3.99	1.26	2.83	0.16	0.12	2.51	**-0.04**	**<0.001**
Midwest	ARIMA (0,1,2)	-10.39	28.82	31.04	33.36	0.35	0.27	7.19	-0.03	0.301
South	ARIMA (0,0,0)	-29.11	64.64	65.84	68.17	0.81	0.44	7.94	0.02	0.495
Brazil	ARIMA (0,0,0)	-34.72	75.1	76.3	78.64	1.03	0.45	8.14	-0.03	0.479

^a^Log-L: Log-Likelihood

^b^AIC: Akaike Information Criterion;

^c^AICc: Akaike Information Corrected Criterion

^d^BIC: Bayesian Information Criterion

^e^RMSE: Root Mean Square Error.

^f^MAE: Mean Absolute Error

^g^MAPE: Mean Absolute Percentage Error

^h^Coef.: Coeficient

## Discussion

In this interrupted time series study involving all the 110,162 deaths due to oral cancer registered in the official Brazilian health system from 1996 to 2019, we observed that OCMR trends are not uniform across Brazilian regions. Besides, NOHP was not associated with a reduction in OCMR in Brazil, except for the Southeast.

### Series decomposition, annual percentage change, and model estimation

In time trend analysis using the Prais-Winsten technique, OCMR tended to increase in the North, Northeast, and Midwest. These results agreed with another work, in which rates for potential years of life lost to this disease increased over time [21]. The results may also reflect an improvement in the reporting and monitoring of deaths from oral cancer in the SIM over time [3, 21]. The trend’s behavior and the highest APC may be related to the worse socioeconomic conditions of these regions *vis-à-vis* the South and Southeast. Furthermore, they suggest difficulties in accessing oral health services along the HCN.

The stable behavior for OCMR in the South and Brazil can be seen in other studies [3, 21], even with differences in periods and grouping formats of the anatomical sites. Although the South has the highest percentage of tobacco users (19.0%) [22] and the highest life expectancy [23] among the macro-regions of Brazil, we could assume that a better organization of work processes in PHC in this region [24] and the significant expansion of oral health services in PHC [25] may be contributing to the static OCMR in this region. In turn, the downward trend in OCMR for the Southeast may probably reflect the better quality of oral health services in this region, especially those of medium and high complexity, which allows for earlier diagnosis and treatment to be started promptly, corroborating the findings of other studies [3, 5, 21].

### The intervention’s effect

In interrupted series analysis, the NOHP was associated with an increased OCMR in the North and a decrease in the Southeast. We raised three hypotheses to explain these unusual results. Firstly, the policy, along with other initiatives, has contributed to improving the process of registering deaths in the SIM, especially in areas with the worst coverage of the system, such as in the North region of the country [26]. Thus, the increased OCMR after NOHP in the North region may mean an improvement in registration instead of more deaths. A previous study revealed the incompleteness of databases on the incidence, distribution, and clinical characteristics of oral cavity and oropharyngeal cancer in Latin America [27], in addition to the fact that, in Brazil, official data are probably underestimated [26, 27]. The monitoring of the disease by clinical staging and the time between the clinical diagnosis and the beginning of the treatment started only after Law N° 12.732/2012 [28] and feeding the Panel-Oncology panel is quite recent [29]. In addition, it is important to mention that oral cancer in Brazil is not a notifiable disease, so managers cannot adequately access data to support decision-making promptly.

Secondly, the non-reduction of OCMR, as expected after 15 years of the policy, may indicate that the care model in Brazil has not been effectively modified, that is, there are still challenges in the proper implementation of the NOHP. Some challenges are related to completeness; extension and improvement of care; integrated teamwork; working conditions; planning, monitoring, and evaluation of actions; stimulating people’s participation and social control; intersectoral actions; universal access to services; and funding [30–32]. Ideally, the implementation of this policy should reduce OCMR in all macro-regions. However, the NOHP did not affect the Northeast, South, Midwest, and Brazil mortality rates, suggesting that the NOHP is not adequately implemented throughout the different national territories and that the oral health care model in Brazil is not capable of effectively impacting health indicators [30]. Although the NOHP is national, its implementation process is quite complex due to social inequalities, territorialization, the fact that the praxis of this policy occurs in the municipality, and that Brazil has more than five thousand municipalities. Therefore, universalizing and municipalizing access to oral health services is a significant challenge.

According to the NOHP, the biopsy of potentially malignant lesions is a procedure included in the PHC dentist’s activities in Brazil, and Stomatology is a mandatory specialty within the CEOs. However, biopsy is rarely performed in PHC and not all CEOs in Brazil performed biopsy (82%), case registration (71.2%), and referral to anatomopathological study (86%), especially in the North and Northeast [33]. These aspects may evidence deficiencies in dentists’ university education, in which educational actions are behaviorist, geared to specific age groups (5–12 years), dental caries control, and weak teaching-service integration [24, 34, 35]. Furthermore, strategies such as the active search for cases in the territory and intersectoral work must still be strengthened [34]. Some important Brazilian legal frameworks were only published in recent years, such as the Law Nº 12.732/2012 [28] and the National Policy for Cancer Prevention and Control [36]. Therefore, NOHP’s intersectionality with other health policies is still under construction. Nonetheless, NOHP’s implementation may be hampered by the ongoing oral health definancing process in the SUS due to the enactment of the Constitutional Amendment Nº 95 in 2016 [37].

Thirdly, we resorted to the three-delay model [38] to hypothesize that a combination of factors may be necessary to reduce OCMR. This model provides a useful framework to examine factors influencing the timeliness of care. The first delay explores socioeconomic and cultural factors influencing a person’s decision-making to seek care. The second delay is mainly influenced by structural factors necessary for reaching the healthcare facility. The third delay describes factors at the healthcare facility such as the availability and quality of the services.

Several factors may have favored the implementation of a differentiated care model for NOHP in the Brazilian Southeast, as we observed a decrease in OCMR after implementing the NOHP in this region. Specifically, these factors include higher education and income, and socioeconomic development in general, associated with good conditions of urban infrastructure, as well as the offer of qualified services in an integrated NHC. This finding aligns with trends previously observed in OCMR [3] and potential years of life lost in the Southeast [21].

Possibly, the increased availability of human and physical resources–such as the expansion of establishments offering oral health services–does not necessarily reflect effective access, corroborating previous findings [32]. Therefore, according to the Andersen and Newman model [39], this type of access depends on the external context, health system characteristics, individual factors, and health needs. These results may also reflect a late inclusion of the oral health team in the PHC [33] and difficulties operationalizing interprofessional work to operate in a network in most of Brazil [30, 32].

Thus, it is necessary to invest in changing work processes, which do not seem to be happening effectively throughout the national territory, even after the NOHP [31]. Furthermore, Brazil displays an incomplete epidemiological transition setting. So, the NHC needs to be organized to deal with acute and chronic conditions. However, the Unified Health System (SUS) operationalization is still very focused on meeting the acute conditions; and, perhaps, the Southeast region, more developed in Brazil, which is a reference for high-complexity treatment, is the best prepared to deal with oral cancer care, as identified in a study that analyzed the adequacy of work processes for oral cancer care [40].

### Limitations and strengths

This work has some limitations, such as the possible underreporting of oral cancer deaths. However, these are the official data from the death notification systems in Brazil; and there is evidence of the suitability of this health information system, which has been improving over time, representing a useful and valid monitoring system in Brazil [20]. Besides, we analyzed annual time series with relatively few points over the time–period available in the SIM during the validity of the ICD-10. However, the low monthly distribution of oral cancer deaths and the absence of information regarding sex and age for death records in the SIM publicly available would hamper rate standardization. Our results cannot be imputed to the individual level, given the possibility of ecological fallacy.

However, this paper also has strengths as the OCMR were standardized by gender and age. The time series included the pre- (1996–2004) and post-NOHP (up to 2019). Also, using the more robust ARIMA method allows for working better with non-stationary series [14]. We did not identify studies that evaluated the relationship between the implementation of the NOHP and the OCMR, which reinforces the unprecedented results in this work. Conducting a time series study is an analytical tool that significantly contributes to health managers’ work in the surveillance of OCMR, the evaluation of health interventions and policies, directing the activities to be developed, and the best way to distribute and allocate health resources [12, 14].

## Conclusions

The implementation of the NOHP in 2004, with the expanded access to primary and specialized dental services in the country, has not yet managed to curb OCMR in Brazil. Although there has been observed an association of the policy with reduced mortality in the Southeast and an increase in the North region, it probably expresses an improvement in the quality of data recording rather than a direct effect. That is, even after 15 years, the Brazilian NOHP is not being effective in controlling OCMR. We recommend NOHP review, strengthening the oral HCN, incorporating oral cancer as a notifiable disease, adopting strategies for prevention, screening, and referral by the PHC, and providing opportunities for early treatment of the disease in Brazil, which may be also useful for other low- and middle-income countries contexts.

## Supporting information

S1 File (XLSX)
